# Unmasking Brugada syndrome: a case report of diagnostic oversights

**DOI:** 10.1093/ehjcr/ytag113

**Published:** 2026-03-18

**Authors:** Juan C Ibarrola-Pena, Rafael Garcia-Ramos, Enrique Paredes-Gutierrez, Gerardo Pozas-Garza, Erasmo De la Pena-Almaguer

**Affiliations:** Tecnologico de monterrey, Escuela de Medicina y Ciencias de la Salud, Av Morones Prieto 3000, Monterrey, Nuevo Leon CP 64710, Mexico; Instituto de Cardiología y Medicina Vascular, TecSalud, Escuela de Medicina y Ciencias de la Salud, Tecnológico de Monterrey, Batallón de San Patricio 112, Real de San Agustin, San Pedro Garza Garcia, Nuevo Leon 66278, Mexico; Tecnologico de monterrey, Escuela de Medicina y Ciencias de la Salud, Av Morones Prieto 3000, Monterrey, Nuevo Leon CP 64710, Mexico; Tecnologico de monterrey, Escuela de Medicina y Ciencias de la Salud, Av Morones Prieto 3000, Monterrey, Nuevo Leon CP 64710, Mexico; Tecnologico de monterrey, Escuela de Medicina y Ciencias de la Salud, Av Morones Prieto 3000, Monterrey, Nuevo Leon CP 64710, Mexico; Instituto de Cardiología y Medicina Vascular, TecSalud, Escuela de Medicina y Ciencias de la Salud, Tecnológico de Monterrey, Batallón de San Patricio 112, Real de San Agustin, San Pedro Garza Garcia, Nuevo Leon 66278, Mexico; Tecnologico de monterrey, Escuela de Medicina y Ciencias de la Salud, Av Morones Prieto 3000, Monterrey, Nuevo Leon CP 64710, Mexico; Instituto de Cardiología y Medicina Vascular, TecSalud, Escuela de Medicina y Ciencias de la Salud, Tecnológico de Monterrey, Batallón de San Patricio 112, Real de San Agustin, San Pedro Garza Garcia, Nuevo Leon 66278, Mexico

**Keywords:** Brugada syndrome, Syncope, Sudden cardiac death, Ventricular tachycardia, Subcutaneous ICD, Case report

## Abstract

**Background:**

Brugada syndrome (BrS) is an unusual cardiac channelopathy associated with an increased risk of ventricular fibrillation (VF) and sudden cardiac death (SCD), highlighting the critical importance of early diagnosis.

**Case summary:**

A 22-year-old male patient presented with recurrent episodes of syncope, palpitations, and dyspnoea. Initial electrocardiograms (ECGs) showing a Brugada Type 1 pattern were misinterpreted as incomplete right bundle branch blocks. The patient was erroneously diagnosed with epilepsy due to misinterpretation of his syncope episodes as seizures. After further investigations, including a propafenone provocation test and cardiac magnetic resonance imaging (MRI), BrS was confirmed. An electrophysiologic study showed no inducibility of ventricular tachycardia (VT) or VF, and a subcutaneous implantable cardioverter-defibrillator (S-ICD) was implanted for the prevention of SCD. The patient’s recovery was successful and uncomplicated.

**Discussion:**

Brugada syndrome patients usually present with SCD, syncope, or severe arrhythmias. Syncope is the most common clinical presentation and identifies patients who could benefit from ICD. Only the Type 1 Brugada pattern is diagnostic, either spontaneous, unmasked by high precordial leads, or by sodium channel inhibition. Implantable cardioverter-defibrillator placement is indicated to prevent SCD. A subcutaneous defibrillator should be considered if the patient has no need for anti-bradyarrhythmia pacing.

**Conclusion:**

Early recognition of BrS in patients with syncope is critical, especially for first-line providers interpreting ECGs, as it may prevent SCD.

Learning points

- Brugada syndrome may present with syncope, sudden cardiac death, or malignant arrhythmias; recognizing high-risk patients is crucial for timely ICD implantation.
- Early identification of Type 1 Brugada electrocardiogram pattern is essential, as misinterpretation may delay life-saving therapy in patients with recurrent syncope.

## Introduction

First described in 1992, Brugada syndrome (BrS) is a rare but potentially life-threatening cardiac channelopathy. It primarily affects men and has a worldwide distribution. Although genetic variants are not present in all cases, the syndrome is associated with mutations in the SCN5A gene, which encodes the cardiac sodium channel. Brugada syndrome is characterized by a distinctive electrocardiogram (ECG) pattern, which can be found either spontaneously or by provocation with sodium channel blockers. Clinical manifestations vary, from asymptomatic cases to life-threatening ventricular fibrillation (VF), and syncope is a frequent indicator of increased arrhythmic risk. Brugada syndrome has been linked to 4%–12% of all sudden cardiac deaths and accounts for about 20% of SCDs in structurally normal hearts.^1^ Given the variable clinical presentation and possible misinterpretation of ECG findings, BrS remains underdiagnosed. Therefore, early and accurate diagnosis is crucial to implement appropriate therapeutic strategies that can be life-saving. In this report, we present the case of a 22-year-old Latino man with a history of recurrent syncope and persistent palpitations who was initially misdiagnosed with epilepsy due to a misinterpretation of his symptoms and ECG.

## Summary figure

- *2 years before admission*: First ER visit for dyspnoea, palpitations, and diaphoresis. Electrocardiogram showed a Type 1 Brugada pattern misinterpreted as incomplete RBBB. Discharged with symptomatic treatment.
- *1 month later*: Second ER visit after syncope while driving with seizure-like movements. Electrocardiogram again showed Type 1 Brugada pattern, misread as incomplete RBBB. Diagnosed with epilepsy and treated conservatively.
- *1 year before admission*: Third ER visit for recurrent syncope. Neurologic work-up [electroencephalogram (EEG) and brain MRI] was unremarkable, and Holter monitoring showed only sinus arrhythmia without evidence of a Brugada pattern.
- *Day 1 (current admission)*: Presented with palpitations, dyspnoea, and diaphoresis. Electrocardiogram showed sinus arrhythmia; retrospective review confirmed previous Brugada Type 1 pattern.
- *Day 2–3*: High precordial ECG leads and propafenone provocation test confirmed Type 1 Brugada pattern. Negative tilt test. Cardiac MRI: 2% fibrosis in RVOT and anterior septum.
- *Day 4*: Electrophysiology study: no VT/VF inducibility. Subcutaneous ICD implanted successfully.
- *Day 8*: Discharged after 3 days of monitoring. No complications.
- *Day 18*: Follow-up visit: full recovery, resumed daily activities.
- *2 years after admission*: Follow-up visit: full recovery, no episodes of syncope, no inappropriate ICD shocks, and no documented VT/VF episodes.

## Case

**Figure1:**
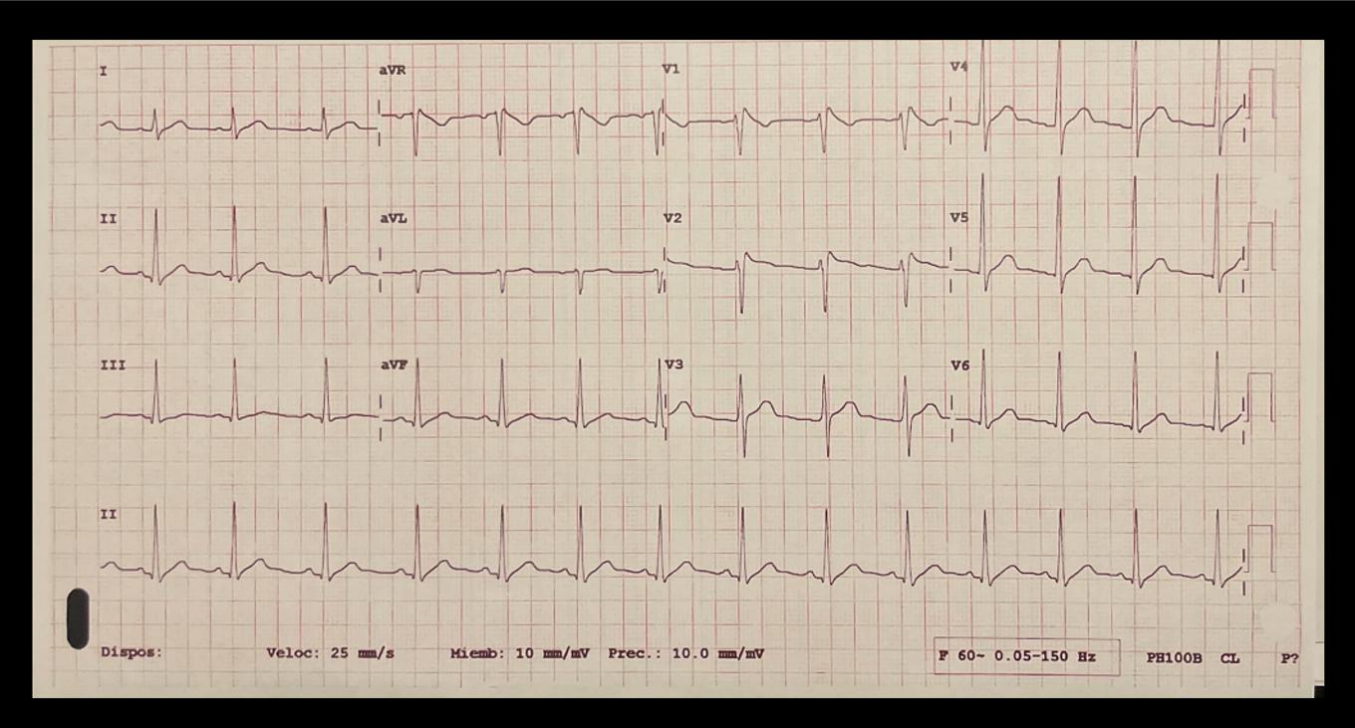
Electrocardiogram showing Brugada type 1 pattern in V1 and V2 incorrectly diagnosed as a right bundle branch block.

A 22-year-old Latin male with a history of asthma, attention-deficit/hyperactivity disorder, and generalized anxiety disorder (GAD) is described. He was treated with fluticasone propionate and salmeterol for asthma and with methylphenidate and fluoxetine for his neuropsychiatric conditions. His family medical history revealed that his grandfather likely died from possible sudden cardiac death (SCD), and his father underwent a cardiac ablation for an unspecified arrhythmia in his 30s. Two years ago, he presented to the emergency room (ER) with dyspnoea, palpitations, and diaphoresis. The initial ECG displayed a Brugada Type 1 pattern, misinterpreted as an incomplete right bundle branch block (RBBB) (*Figure 1*), leading to symptomatic treatment and discharge. Thirty days later, he experienced syncope while driving, with witnesses reporting seizure-like movements. He was neurologically intact, revealing only a left humerus fracture, treated conservatively. Once again, the ECG displayed a Brugada Type 1 pattern, but it was misinterpreted again as an incomplete RBBB. A year later, a second syncope occurred under similar circumstances. He underwent evaluation with an EEG, brain MRI, and Holter monitor, which yielded no significant findings; the ECG indicated only sinus arrhythmia. At this point, the patient was taking levetiracetam for seizure management.

**Figure 2:**
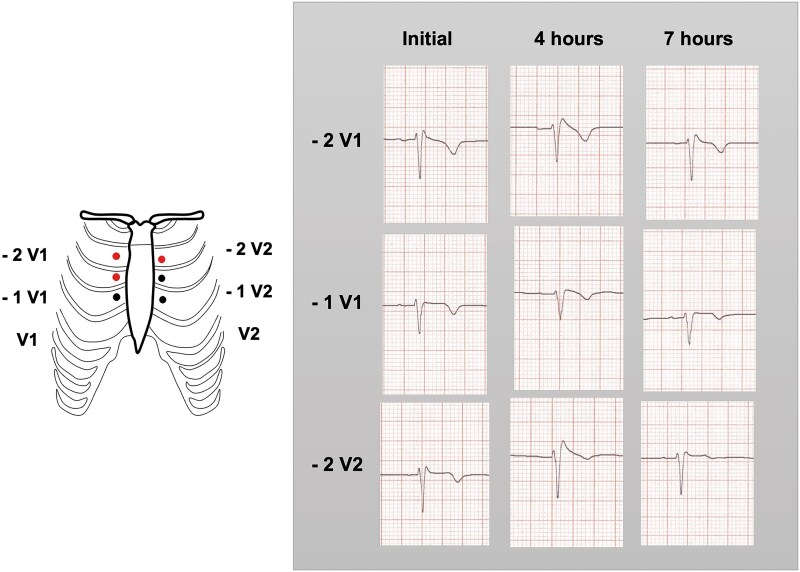
High precordial leads and propafenone provocation test unmasking the Brugada pattern.

During the fourth ER visit for persistent palpitations, dyspnoea, and diaphoresis, a follow-up ECG showed sinus arrhythmia. A retrospective review of prior ECGs prompted initiation of the BrS protocol. High precordial leads were recorded to exclude improper electrode placement, revealing an inconclusive terminal R-wave. Due to limited access to intravenous class I antiarrhythmics in Latin America, a propafenone challenge was conducted, which unmasked a type 1 pattern in −1V1, −2V1 and −2V2 leads (*Figure 2*). To rule out neurocardiogenic syncope, we performed a tilt table test with isosorbide, showing sinus bradycardia and hypertension, excluding that aetiology. Cardiac MRI revealed normal ventricular function, with areas of diffuse myocardial fibrosis. With a full-width maximum method, 2% of fibrosis was averaged base, extending to the anterior septum and the right ventricular outflow tract (RVOT).

Following the confirmation of BrS, the patient and his relatives received genetic counselling. The genetic panel was negative for known pathogenic variants associated with BrS. No results were available for the father, and testing could not be performed on the grandfather due to the time elapsed since his death.

**Figure 3:**
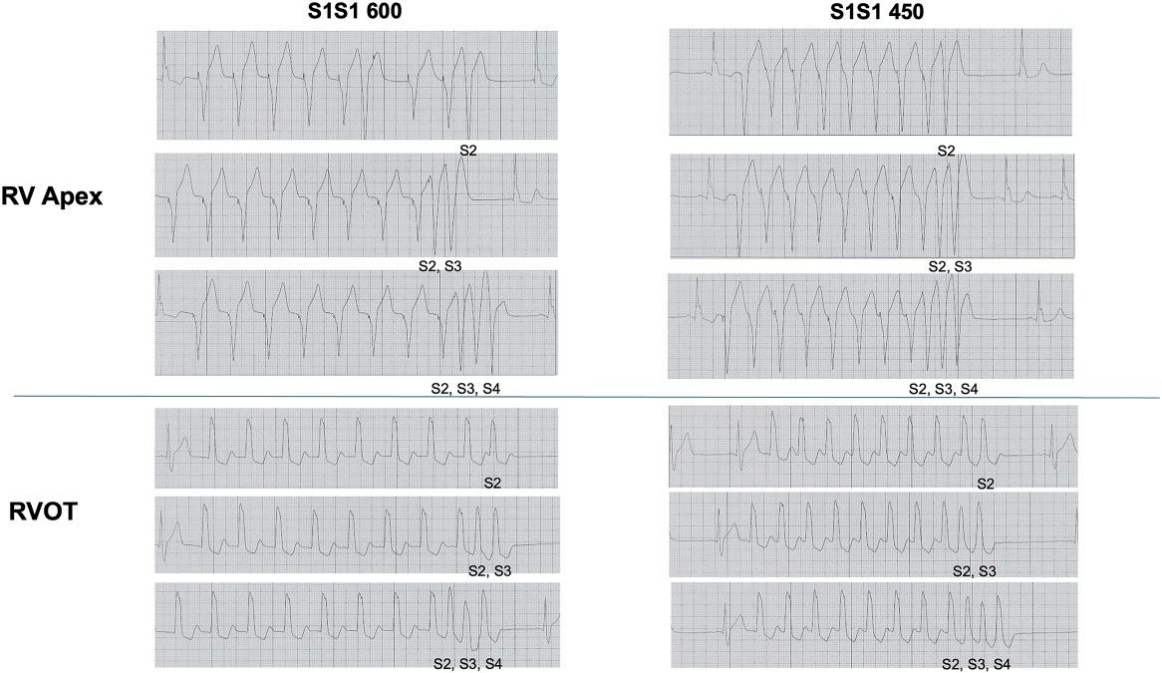
Ventricular programmed electrical stimulation showing no VT/VF inducibility.

An electrophysiologic study (EPS) was performed to evaluate ventricular tachycardia (VT) or ventricular fibrillation (VF) inducibility and to assess sinus and atrioventricular node function. Programmed ventricular stimulation was performed using two basic drive cycles (S1S1) of 600 and 450 ms, with up to three extrastimuli delivered at the right ventricular apex and RVOT. The ventricular refractory period was 230 ms, and no VT or VF was induced. Sinus and atrioventricular node function were normal (*Figure 3*). These findings did not influence clinical decision-making, as the indication for implantable cardioverter-defibrillator (ICD) implantation was primarily based on the patient’s recurrent unexplained syncope and a documented Type 1 Brugada pattern, in accordance with current guideline recommendations.

**Figure 4:**
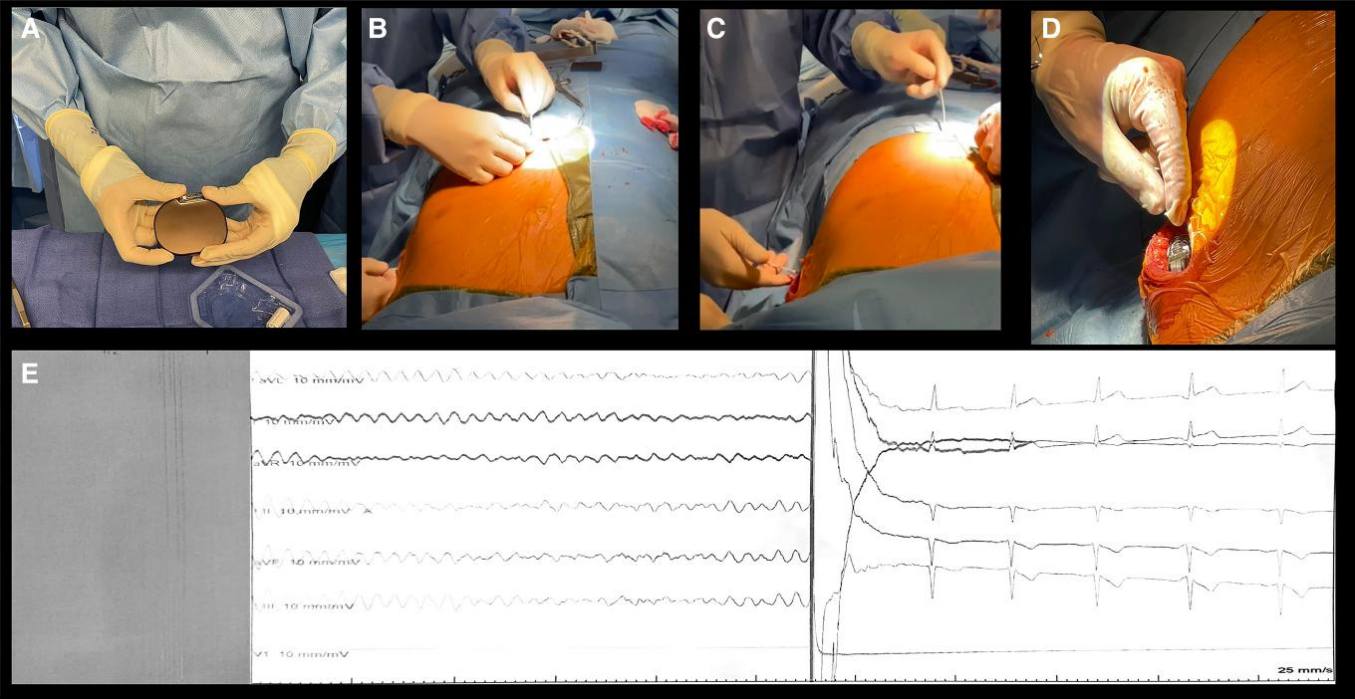
Sequence of subcutaneous implantable cardioverter-defibrillator placement (*A–D*) X-ray showing proper electrode and device position. (*E*) The induced ventricular fibrillation is terminated and sinus rhythm is restored with a shock delivered by the subcutaneous defibrillator.

Given the patient's specific characteristics, a subcutaneous implantable cardioverter-defibrillator (S-ICD) was implanted using a two-incision technique along the left parasternal line. During testing, VF was induced with 50 Hz stimulation and successfully terminated by a 65-J shock. The shock zone was programmed for rates above 220 b.p.m. with post-shock stimulation. The procedure was uneventful, and the patient was discharged after 3 days (*Figure 4*).

After 2 years of follow-up, the patient reported full recovery, with no pain or any infection-related symptoms. He received counselling on warning signs, promptly addressing fever, and instructions to avoid medications that could trigger BrS. A couple of months after his ICD implant, he has fully adapted to the device, returned to university, and resumed his daily exercise routine. The patient has not experienced further syncopal episodes, inappropriate shocks, or documented VT/VF. A semi-annual consultation is planned, followed by annual surveillance.

## Discussion

This case emphasizes the importance of a thorough clinical history and the ability of first responders to recognize critical ECG findings. Despite the presence of sinus arrhythmia on the ECG at the current presentation, several aspects of the patient’s prior history raised concern for an underlying arrhythmic syndrome and prompted a retrospective review of previous ECGs. During evaluation in our Cardiodiagnostics Department, the unusually high number of ECGs performed in a patient of such young age triggered a review of prior tracings and a detailed reassessment of his clinical history. Brugada syndrome is diagnosed by a Type 1 Brugada pattern, defined by >2 mV coved ST-elevation and negative T-wave in lead V1 or V2, regardless of symptoms and in the absence of other cardiac conditions that could explain the pattern.^2^ Given that approximately one-third of patients with BrS present with syncope, first responders should maintain a strong suspicion when encountering such symptoms. Other associated symptoms, such as nocturnal agonal respirations, should be considered, as they may indicate an increased risk of cardiac events.^3^

The patient was taking fluoxetine for GAD and levetiracetam for seizure management. Levetiracetam has not been associated with exacerbation of the Brugada pattern or cardiovascular syncope. However, SSRIs like fluoxetine are known to potentially unmask or exacerbate the Brugada pattern, which was considered in the overall management plan.

Repeated misinterpretation of the ECGs in this case raised concerns about the potential for misdiagnosis that may occur daily. In 2016, Gottschalk *et al*.^4^ reported a high rate of ECG misinterpretation even among cardiologists. Their results showed that the mean accuracy for recognizing Brugada patterns was 43 ± 33%, for diagnosing BrS was 63 ± 34%, and the overall accuracy was 53 ± 33%.^4^ If such results were obtained by trained cardiologists, similar or even lower accuracy rates can be expected among first responders, highlighting serious concerns about the potential complications associated with this syndrome.

Cardiac MRI in our patient demonstrated minimal diffuse fibrosis (2%) involving the basal anterior septum and RVOT. In the study by Bastiaenen *et al.*,^5^ LGE was identified in only 8% of BrS patients, predominantly localized to the left ventricular midwall or RV insertion points, with no association with arrhythmic outcomes. These data indicate that fibrosis detected by CMR is uncommon and its clinical significance in BrS remains uncertain. Therefore, the minimal fibrosis observed in our patient may represent a non-specific finding rather than a structural correlate of the Brugada phenotype.

The decision to proceed with ICD implantation was based on established high-risk clinical features rather than on documented aborted cardiac arrest or sustained ventricular arrhythmias. The patient presented with recurrent unexplained syncope of presumed arrhythmic origin and a documented spontaneous Type 1 Brugada pattern, both of which are recognized markers of increased risk for sudden cardiac death. According to the HiRO consensus handbook on inherited arrhythmia syndromes, symptomatic patients with a spontaneous Type 1 Brugada pattern and arrhythmogenic syncope are considered at high risk and should be considered for ICD therapy.^6^ This approach is also consistent with the 2022 ESC Guidelines,^3^ which recommend ICD implantation in selected high-risk BrS patients based on clinical presentation and ECG findings.

The ESC 2022 Guideline recommends ICD implantation in BrS and aborted cardiac arrest (CA) or spontaneous sustained ventricular arrhythmia (VA) and considered in the presence of Type 1 Brugada pattern (Class 2A), given the recurrence of VF among patients presenting CA is 48% at 10 years.^3^ In recent years, there has been ongoing controversy regarding the use of transvenous implantable cardioverter-defibrillator (TV-ICDs) vs. S-ICDs. While TV-ICD remains used, it has certain advantages, especially for young patients with BrS who have a long life expectancy. Some of the main concerns include higher defibrillation thresholds, which are inappropriate, particularly due to T-wave oversensing, resulting from relative changes in the R-wave to T-wave ratio that lead to overcounting and lead to failure. Subcutaneous ICD has emerged as an alternative, as it mitigates complications related to lead placement, infection risk, and difficulties associated with system extraction in patients who typically do not require ventricular pacing. However, a significant limitation of S-ICD is its inability to provide atrial sensing, which is essential for accurately identifying AF.^7^ This is particularly relevant, as Sarkozy *et al*.^8^ have shown that up to 14% of asymptomatic AF cases can result in inappropriate shocks. To optimize patient selection and minimize complications, careful screening—including evaluations during exercise testing, drug provocation testing, and QRS voltage assessment—should be considered, along with strategies such as pharmacological heart rate control or activity modification in selected cases.^9^

In retrospect, the patient’s management was consistent with current recommendations for BrS. According to the HiRO consensus on inherited arrhythmia syndromes, symptomatic patients with a Type 1 Brugada pattern should undergo a 12-lead ECG, detailed family history assessment, genetic testing, and consideration of an electrophysiological study.^6^ These steps were appropriately followed in this case, supporting accurate diagnosis and appropriate treatment with subcutaneous ICD implantation.

## Conclusions

Brugada syndrome patients usually present SCD, syncope, or severe arrhythmias. Syncope is the most common clinical presentation and identifies patients who could benefit from ICD. First responders need to recognize the relationship between syncope and BrS and approach ECG interpretation with heightened awareness. Only the Type 1 Brugada pattern is diagnostic, either spontaneous, unmasked by high precordial leads, or by sodium channel inhibition. Implantable cardioverter-defibrillator placement is indicated to prevent SCD. A subcutaneous defibrillator should be considered if the patient has no anti-bradyarrhythmia pacing.

## Data Availability

No new data were generated or analysed in support of this research.

## Lead author biography

Dr Juan Carlos Ibarrola-Pena is a third-year cardiology resident at the Institute of Cardiology and Vascular Medicine at TecSalud in Monterrey, Mexico. He received his medical degree from Universidad Autónoma de Guadalajara. Dr Ibarrola-Pena is also a member of the InkTeam research group. His primary areas of interest include the diagnosis and management of arrhythmias, as well as advanced techniques for treating cardiac rhythm disorders.

## Notes

**Consent:** Written informed consent for publication of this case report, including images and associated text, was obtained from the patient in line with COPE guidance.
